# Annotations

**Published:** 1902-03-08

**Authors:** 


### ANNOTATIONS.

### Boarding Out.

The State Children's Association again have issued a plea for the boarding out of pauper children. Most people who have given much attention to the question are of opinion that it is better for children who are the wards of the State to be brought up in a family, away from the associations of the workhouse, rather than be made from childhood to feel that they were mere inmates of an institution, where the simple necessity of maintaining discipline render inevitable the suppression of all individuality. Such a system involves children being sent into the outer world, when they are of an age to become self-supporting, with all their experience of life to learn and all their mistakes to make. The variety and intimacy of home life, the more or less unfettered intercourse with outsiders, form a part of education for which institutions offer but a poor substitute. Abroad, and in some of our own colonies, this fact is so thoroughly accepted that only a small proportion of pauper children are kept within the walls of the union. Thus, France boards out all its dependent children, and Germany all its pauper girls. In Russia about 90 per cent. of the children are so dealt with, and in Switzerland about 74 per cent. Of Scotch pauper children 84 per cent. are boarded out, and in Queensland nearly 100 per cent. are kept outside the workhouse. And England boards out only about 5 per cent. of its dependent children. Boarding out has the recommendation of cheapness, a boarded-out child costing on an average £13 6s. 8d. a year, as against the £29 5s. 6d. which is the average cost of maintenance of a child in one of the large Metropolitan barrack schools. The difficulty of boarding out all dependent children is that of finding a sufficient number of suitable homes; and the great number of children that have to be maintained by most of our large unions makes this a serious matter. Inspectors, by preference women inspectors, are needed to pay pretty frequent surprise visits to the home were the child is living, in order to prevent hypocritical people ill-treating their wards when alone with them, while keeping a fair face to the authorities. But it is possible to get voluntary workers to undertake this duty in many instances, and the benefit to the children of interesting a kind-hearted man or woman in their fate, may be, and very often is, a great and permanent one.

### "Farthing Land."

A Scotchman who has gone to London has been asked by one or more of his fellow-countrymen if life there is cheaper than in the north. His answer, as given in the *Glasgow Evening News* is, practically, "that depends." If the southward-bound Scot expects to live in as good a house, in as good a locality, as he did at home, the probabilities are that he will have to pay more for these amenities. But, as the newsman doubtless knows, there are Scots who don't expect all that—at least to begin with. For these, in the earlier stages of their metropolitan manvantara, he recommends Dorset Street, Poplar, where things are sold by the farthing's worth, and a savoury and sustaining meal can be had for a penny. The *menu* of a Dorset Street restaurant would enlighten one as to its possibilities. A satisfying breakfast is there made up of farthings'-worths. Bill of fare:—bloater, ¼d.; margarine, ¼d.; bread, ¼d.; coffee, ¼d. Or for the bloater and margarine the hungrier may substitute a farthing rasher and a farthing kipper. For dinner the plutocrat of Dorset Street may have three ounces of steak or chop, costing a half-penny, two potatoes at a farthing, and a farthing's worth of bread. All which shows that those kind-hearted persons who draw out menus for the poor, and then lament that at the wages they earn they cannot afford them, should consult the market prices in Dorset Street before they cry out in despair. Not that everything is cheaper in Dorset Street than elsewhere. You can buy a farthing's worth of coal, it is true, but for that you get only a pound of the worst quality of fuel, and the price works out at something like £2 per ton. But even so, the farthing's worth is something of a boon to people who will never have enough money in hand at one time to buy a ton of coal, nor a place in which to store it if they had. But we mention this to deter the housekeepers of London from rushing in a body to do their marketing in Poplar, and thereby raising the prices against the poor souls who live there. Perhaps, however, a visit to the East End and the sight of the shops where such good bargains are to be had would cure all but the most determined seekers after cheapness.

### An Amazing Hospital Scandal.

The inhabitants of Portsmouth and the district have good grounds for grave indignation at the scandals disclosed at the annual meeting of the Royal Portsmouth, Portsea, and Gosport Hospital. We believe it is not too severe a statement to make that the Royal Portsmouth Hospital is probably the only hospital in this country of which it could be said, at the present day, that the hospital moneys had been misappropriated, that the fact was not stated in the report of the committee to the governors, and that the auditors could only certify the accounts as correct subject to their full report of a grave character. This report recorded defalcations by an official, upwards of £100 unaccounted for, £10 short paid into the bank, and the acknowledgment of subscriptions in the report which had not been received by the hospital. It further appeared that in the statement of liabilities and assets several items had been altered or suppressed, and the auditor emphatically protested against this alteration of the accounts, after he had signed them, without his sanction or knowledge. Had the uniform system of accounts been adopted at Portsmouth, these grave abuses could not have occurred. In the result, the report and balance-sheet were referred back to the committee by the governors, and a sub-committee was appointed to inquire into matters and report to a special meeting of governors. The foregoing statements made at the annual meeting of the Royal Portsmouth Hospital, as reported in the *Portsmouth Evening News* of February 26th last, if true, are so serious that in our view the committee under whose management these abuses were permitted to occur, should be replaced by new men. Indeed the present committee may possibly be moved for their own sakes to resign on public grounds, and so enable the essential reforms to be carried out promptly and efficiently.

